# The Beat

**DOI:** 10.1289/ehp.120-a423

**Published:** 2012-11-01

**Authors:** Erin E. Dooley

## Green Chemistry with Coffeehouse Waste

Investigators from the City University of Hong Kong reported at the 244th meeting of the American Chemical Society on a new process that could produce bio-based products without diverting crops from food.^^1^^ The process, developed by Carol S.K. Lin with the support of Starbucks Hong Kong, involves blending waste food such as used coffee grounds and stale baked goods with a mixture of fungi. Enzymes produced by the fungi break the carbohydrates in the waste into simple sugars that are fermented into succinic acid, a “platform molecule” that can be used to produce plastics, laundry detergents, and medicines.

## EU Publishes Guidelines for Quantifying Noise Exposure

Studies on the health effects of noise exposure have suffered from discrepancies in how such exposure is quantified. In an effort to provide uniform and comparable data across studies, the European Commission’s Joint Research Centre recently released a set of guidelines titled Common Noise Assessment Methods in Europe, or CNOSSOS-EU.^^2^^ The guidelines define common noise indicators and set forth best practices for assessing noise exposures and submitting noise data to Europe’s Electronic Noise Data Reporting Mechanism. These methods will be used across the European Union to help member states comply with the 2002 Environmental Noise Directive,^^3^^ under which countries are required to develop noise maps to monitor and address local noise problems.

**Figure:**
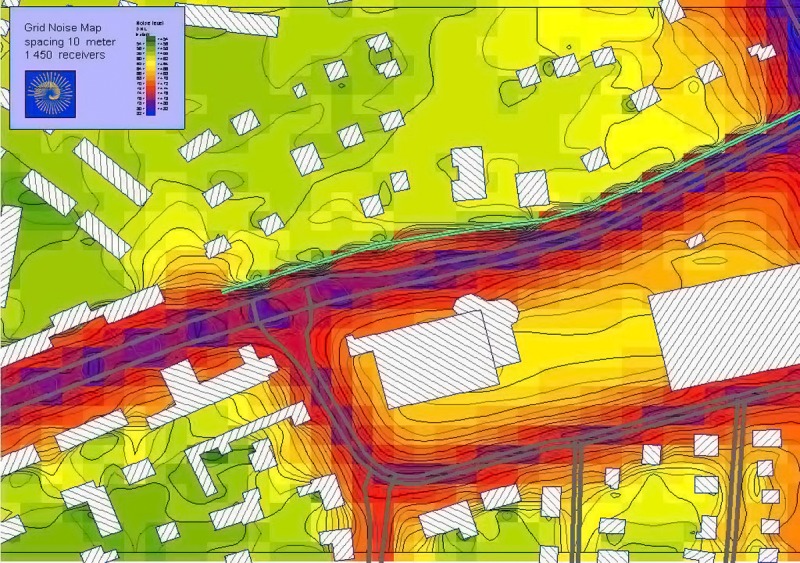
Noise maps depict the distribution of sound through a given area. Navcon Engineering Network

## Canada Reverses Course on Asbestos Listing

In September 2012 Canadian industry minister Christian Paradis announced the country will no longer oppose international efforts to list asbestos under the United Nations’ Rotterdam Convention, which requires that exporters of potentially harmful substances notify importing countries of relevant health hazards.^^4^^ The Canadian Public Health Association applauded the shift, which came months after the Joint Policy Committee of the Societies of Epidemiology issued a statement that called for an end to trade in the mineral.^^5^^ In addition, the newly elected government of Québec has announced it will cancel a Can$58-million loan to revive the Jeffrey Mine, Canada’s sole remaining asbestos mine. Asbestos could be added to the Rotterdam Convention when parties to the convention convene in 2013.

## Atrazine Linked to Birth Defects

In a study of 280 Texan children diagnosed with choanal atresia or stenosis, researchers conclude that these two types of birth defects of the nasal passage may be associated with maternal exposure to atrazine, a suspected endocrine disruptor and the most widely used herbicide in the United States.^^6^^ Mothers in counties with the highest estimated atrazine application had a 79% increased risk of giving birth to a child with one of these defects compared with mothers in counties with the lowest estimated application. The authors, while stressing this did not prove a causal relationship, did emphasize that the results were a good first step in understanding the basis of these birth defects.

## Director Named for New NIH Center

In September 2012 National Institutes of Health (NIH) director Francis Collins announced the appointment of Christopher P. Austin as director of the National Center for Advancing Translational Sciences, the NIH’s newest center.^^7^^ Austin had served as director of the Division of Pre-Clinical Innovation since the center’s launch in December 2011. Austin, a developmental neurogeneticist, earned his medical degree from Harvard University and has been with the NIH since 2002. The goal of the new center is to make translational science more efficient, less expensive, and less risky.^^8^^

## And Finally, Because Dogs Are People, Too . . .

Pets share many environmental exposures with their owners, and like children, their exposures may be higher because they spend more time closer to the ground. Studies have linked exposure to lawn care chemicals with higher risks for canine lymphoma^^9^^ and bladder cancer.^^10^^ The Golden Retriever Lifetime Study is a new study of links between environmental exposures and cancer rates in this breed, which is known to be susceptible to the disease. The study will follow 3,000 young adult dogs throughout their lives.^^11^^ Co-investigator Rodney Page of the Colorado State University Animal Cancer Center likened it to the Nurses’ Health Study and said it will “offer a new set of data with which to evaluate similarities with human exposure data.”^^12^^

**Figure:**
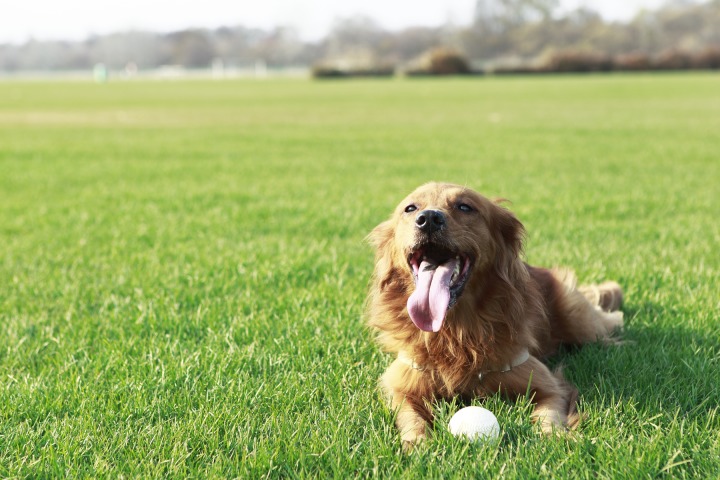
A longitudinal study of golden retriever health may also offer insights into human disease. tomocam/Shutterstock.com
